# Research progress on the impact and mechanisms of helicobacter pylori infection on the efficacy of immunotherapy for gastric cancer

**DOI:** 10.3389/fonc.2025.1674814

**Published:** 2026-01-15

**Authors:** Bin Yao, Chenyu Hou, Weishuai Zhang, Zicheng Bao, Yong Li, Zhidong Zhang

**Affiliations:** The Third Department of Surgery, Fourth Hospital of Hebei Medical University, Shijiazhuang, China

**Keywords:** gastric cancer, gut microbiota, helicobacter pylori, immune checkpoint inhibitors, immunotherapy, PD-1/PD-L1, tumor microenvironment

## Abstract

**Background:**

Helicobacter pylori (H. pylori), recognized as a Group I carcinogen by the World Health Organization, is a key etiological agent in gastric cancer (GC). The majority of GC patients, particularly in China, present at advanced stages with constrained therapeutic options. Tumor immunotherapy, especially immune checkpoint inhibitors targeting the PD-1/PD-L1 axis, has emerged as a promising strategy. However, immunotherapy benefits only a subset of patients. Notably, H. pylori infection plays a significant role in GC and may also influence the efficacy of immunotherapy.

**Main content:**

This review systematically summarizes the role and mechanisms of H. pylori in GC development, progression, and immunotherapy, focusing on the following aspects. Pathogenic mechanisms: H. pylori drives GC development through virulence factors (e.g., CagA, VacA, urease), which induce chronic inflammation, epithelial damage, immune evasion, and remodeling of the tumor microenvironment. Impact on immunotherapy and underlying mechanisms: The clinical efficacy is conflicting, some studies associate H. pylori infection with poor prognosis following immunotherapy, while others to better responses. Proposed mechanisms include PD-L1 upregulation via multiple signaling pathways, modulation of immune cells within the tumor microenvironment, and gut microbiota alterations affecting PD-1/PD-L1 inhibitor efficacy.

**Conclusion:**

H. pylori has a complex influence on GC immunotherapy. Further research is needed to clarify the underlying mechanisms and assess the predictive value of H. pylori testing in clinical practice. Combining microbiome-based strategies with immunotherapy may enable more personalized and effective treatment.

## 1 Introduction

Helicobacter pylori (H. pylori) is the most important pathogen that colonizes the gastric mucosal epithelial cells. It has been classified as a Group I carcinogen by the World Health Organization (WHO), affecting more than 50% of the global population (1). H. pylori infection is the primary cause of chronic gastritis and peptic ulcers, and it is a key risk factor for the development of gastric cancer (GC). It is estimated that 74.5% of GC cases in China can be attributed to H. pylori infection (2). Its pathogenic process spans the entire sequence from chronic inflammation, atrophic gastritis, intestinal metaplasia, and dysplasia to the eventual development of GC.

As a significant global health burden, global statistics from 2020 showed that there were 1.1 million new cases of GC worldwide, ranking fifth among all malignancies and 769,000 deaths, ranking fourth in cancer-related deaths. Due to China’s large population base, GC cases account for nearly half of the global total (3, 4). Furthermore, nearly 70% of GC cases in China are diagnosed at an advanced stage, missing the optimal window for endoscopic or surgical radical treatment. Consequently, treatment relies primarily on systemic anti-tumor drug therapy, leading to suboptimal overall outcomes. The 5-year overall survival (OS) rate is less than 20%, with an extremely poor prognosis (5, 6).

In recent years, tumor immunotherapy, represented by immune checkpoint inhibitors (ICIs), has brought new hope for GC patients. Particularly, programmed cell death protein 1 (PD-1)/programmed death-ligand 1 (PD-L1) inhibitors block immune suppression signals and restore T-cell cytotoxicity against tumor cells. These inhibitors have become an important treatment option for advanced GC (7). However, clinical data show that only 20% of achieve sustained responses to immunotherapy, while a significant proportion show no response or develop resistance, and the exact reasons remain unclear (8). This indicates that the patient’s immune level significantly affects the efficacy of treatment. Considering that H. pylori is one of the key biological factors in GC development, it can shape an immune-suppressive tumor microenvironment (TME) through various pathways (9). It can regulate the host’s innate and adaptive immune responses, thereby affecting the effectiveness of immunotherapy. Additionally, H. pylori can further regulate immune responses by modulating the expression of PD-L1 in host cells and altering the gut microbiota. These mechanisms collectively promote GC immune escape and are likely to significantly influence the efficacy of immunotherapy. This article reviews the impact of H. pylori infection on the efficacy of immunotherapy for GC and the potential mechanisms involved.

The relationship between H. pylori infection and the efficacy of immunotherapy for GC has been examined in several reviews, each approaching the topic from a distinct perspective. For example, some studies have systematically elaborated the core molecular mechanisms by which H. pylori contributes to GC (10, 11); other research has focused on the impact of H. pylori infection on the clinical efficacy of immunotherapy (12, 13). Additionally, certain reviews have addressed the role of H. pylori in immune regulation across multiple cancer types, though their discussion on GC remains insufficiently detailed (14). Meanwhile, specific investigations into the mechanisms by which H. pylori regulates PD-L1 expression and modulates the gut microbiota have been reported (15), and some literature systematically summarize key virulence factors of H. pylori in remodeling of the TME (16). However, most existing studies tend to focus on a single dimension, lacking cross-mechanistic and multi-level integrated analysis.

Building on this foundation, the present review aims to provide a more systematic, updated, and integrative perspective, which is reflected in the following aspects. First, it systematically synthesizes recent conflicting clinical evidence regarding the dual role of H. pylori in immunotherapy, consolidating findings that suggest both potential adverse and beneficial effects. Second, it constructs an interconnected mechanistic framework that integrates H. pylori virulence factors, multi-pathway regulation of PD-L1, dynamic changes in the TME, and interactions with the gut microbiota into a coherent logical system. Third, it incorporates the latest clinical advances, such as H. pylori-mediated induction of tertiary lymphoid structures (TLS), exosome-driven signaling, and gut microbiota metabolites. These aspects were not adequately discussed in previous reviews. Furthermore, it also highlights the potential for clinical translation, explores the possibility of using the H. pylori infection status as a marker for patient stratification, and proposes a translational direction of optimizing treatment strategies by intervening in H. pylori-related immune pathways. Therefore, this review not only consolidates and updates existing knowledge, but also offers a forward-looking perspective with mechanism guidance and evidence support for personalized immunotherapy for H. pylori-related GC.

## 2 The pathogenic mechanisms of H. pylori in gastric cancer development

H. pylori is a Gram-negative, spiral-shaped, microaerophilic bacterium with flagella that colonizes the surface of the human gastric mucosa (17). The infection is a major etiological factor of various gastric mucosal diseases, including chronic non-atrophic gastritis, atrophic gastritis, intestinal metaplasia, dysplasia, and GC (18). The WHO has classified H. pylori as a Group I carcinogen, explicitly stating that chronic infection causes at least 75% of GC cases, with infection increasing the lifetime risk of GC by approximately 1%-5% (1, 19). Given its significant carcinogenicity, the pathogenic mechanisms, carcinogenic pathways, and immune escape strategies of H. pylori have emerged as a core focus of current research.

It is worth noting that H. pylori infection can also lead to other diseases, including gastritis, gastric ulcers, and gastric mucosa-associated lymphoid tissue (MALT) lymphoma (20). Among these, gastric MALT lymphoma demonstrates a particularly strong association with H. pylori infection. This relationship was first reported in 1991 and has since been corroborated by extensive epidemiological and histopathological evidence. Approximately 90% of gastric MALT lymphoma patients are infected with H. pylori (21). Based on this well-established causal link, H. pylori eradication has become the first-line treatment for early-stage gastric MALT lymphoma. Studies have demonstrated that H. pylori plays a crucial role in the pathogenesis of gastric MALT lymphoma. By undergoing H. pylori eradication treatment, 50%-90% of patients in stage I/II_1_ can achieve complete remission (22, 23). Besides gastric pathologies, H. pylori infection is also associated with various extragastric diseases, including cardiovascular, immune, and neurological disorders (24). The common pathophysiological basis of all H. pylori-related diseases, ranging from inflammation to malignancy, lies in its long-term gastric colonization and the consequent chronic inflammation along with persistent alterations in the TME.

H. pylori drives the occurrence and development of GC directly and indirectly through its various virulence factors (**Table 1**). Its primary virulence factors include cytotoxin-associated gene A protein (CagA), vacuolar cytotoxin A (VacA), urease, flagella, and outer membrane proteins (OMPs) (35). CagA, one of the most critical virulence virulence factors, is a 120–140 kDa protein whose polymorphism primarily reflects in differences within its N-terminal domain (36). H. pylori injects CagA into gastric epithelial cells through the type IV secretion system (T4SS) (25). Upon entry into host cells, CagA is phosphorylated at its C-terminal EPIYA motifs by host tyrosine kinases, such as c-SRC and c-ABL (26). The phosphorylated CagA abnormally activates downstream proliferation and survival signaling pathways, thereby participating in the carcinogenesis process (27). Furthermore, CagA can modulate signaling pathways such as STAT3 and NF-κB, promoting the expression of pro-inflammatory cytokines like IL-6 and IL-8, exacerbating local inflammation and immune escape, which further increases the risk of developing GC (28, 29). VacA is a secreted pore-forming toxin that forms channels in host cell membranes, leading to ion efflux and mitochondrial dysfunction, which induces cellular vacuolation, apoptosis, and disruption of the epithelial barrier (30). Regarding immune evasion, VacA can induce T cell apoptosis and inhibit the function of antigen-presenting cells, thereby aiding H. pylori in achieving immune tolerance and long-term colonization (31). Urease and flagella play central roles in bacterial colonization and persistent infection. Urease breaks down urea to produce ammonia, effectively neutralizing gastric acid and creating a microenvironment conducive to H. pylori survival in the highly acidic stomach (32). Flagella give bacteria the ability to move, enabling H. pylori to penetrate the mucus layer and adhere closely to epithelial cells (33). It is worth noting that the urease activity is not limited to colonization. The ammonia it produces can stimulate epithelial cells to release IL-8, thereby recruiting immune cells such as neutrophils. This process exacerbates the inflammatory response and mucosal damage, ultimately fostering a pro-carcinogenic microenvironment (17). Moreover, H. pylori OMPs (such as BabA, SabA, OipA) can specifically recognize receptors on the surface of host epithelial cells, facilitating bacterial adhesion. These OMPs can also induce the production of inflammatory mediators like IL-8, accelerating gastric mucosal inflammatory injury and the process of carcinogenesis (34).

**Table 1:** Mechanisms of H. pylori in promoting gastric cancer via virulence factors.

Virulence factors	Roles of H. pylori	Result
CagA	Injected into gastric epithelial cells through T4SS, and the C-terminal EPIYA motif is phosphorylated by the host kinase (25, 26).	1. Abnormally activates proliferation and survival signaling pathways, contributing to carcinogenesis (27). 2. Regulates the STAT3 and NF-κB pathways, and exacerbate local inflammation and immune evasion (28, 29).
VacA	Forms channels in host cell membranes.	1. Causes ion efflux and mitochondrial dysfunction, leading to cellular vacuolation, apoptosis, and epithelial barrier disruption (30). 2. Induces T-cell apoptosis and inhibits antigen-presenting cell function, aiding immune tolerance and long-term bacterial colonization (31)
Urease	Hydrolyzes urea to produce ammonia (32).	1. Neutralizes gastric acid, creating a survival-conducive microenvironment (32). 2. Ammonia stimulates epithelial IL-8 release, fostering a pro-carcinogenic microenvironment (17).
Flagella	Provide motility to the bacterium (33).	Enables penetration of the mucus layer and close adherence to epithelial cells, facilitating colonization (33).
OMPs	Specifically recognize receptors on host epithelial cell surfaces (34).	Induce production of inflammatory mediators (e.g., IL-8), accelerating gastric mucosal inflammatory injury and carcinogenesis (34).

## 3 Overview of immunotherapy for gastric cancer

Tumor immunotherapy is an emerging treatment strategy that aims to trigger anti-tumor immune responses. It activates immune cells and enhances the immune system’s ability to identify and eliminate tumor cells, thereby inhibiting tumor growth and metastasis. Compared to traditional chemotherapy and radiotherapy, immunotherapy offers higher specificity, more durable efficacy, and relatively milder adverse effects (9). Current main strategies for GC immunotherapy include ICIs, adoptive cell therapy (ACT), chimeric antigen receptor T-cell (CAR-T) therapy, and tumor vaccines (37), with ICIs being the most widely studied. ICIs primarily work by targeting and blocking immune checkpoint proteins expressed on the surface of T cells or tumor cells. This blockade prevents tumor immune evasion, thereby reactivating the host immune system’s ability to eliminate tumor cells and inhibiting tumor progression. Notably, immunotherapy represented by ICIs has shown increasingly prominent efficacy in locally advanced and metastatic GC, with particularly remarkable outcomes observed in tumors exhibiting high expression of PD-L1 (38).

### 3.1 Overview of immune checkpoint inhibitors

Immune checkpoints (ICs) are regulatory proteins in the immune system that typically exist on activated lymphocytes and tumor cell surfaces. These checkpoints help modulate immune tolerance, prevent autoimmunity, and protect tissues from immune attack. They can also inhibit T-cell activation and proliferation, enabling tumor cells to escape immune surveillance. This effect is primarily mediated through the PD-1/PD-L1 pathway, which can inhibit T cell activation, proliferation, and cytokine production (39). ICIs are monoclonal antibodies targeting regulatory immune checkpoint molecules that inhibit T cell activation. ICIs primarily act on immune checkpoints to modulate the activation of T cells in the body’s immune system, generating anti-tumor immune responses to inhibit tumor development.

The ICs in the human body can be categorized into two types: stimulatory checkpoints that promote anti-tumor immunity and inhibitory checkpoints that suppress anti-tumor immunity. The ICIs widely reported in clinical practice primarily target the latter to activate anti-tumor immune responses. Among these, inhibitors of PD-1, PD-L1, and cytotoxic T lymphocyte-associated antigen-4 (CTLA-4) are the most well-established. Studies have shown that these inhibitors can improve OS in patients with melanoma, non-small cell lung cancer (NSCLC), urothelial carcinoma, and other malignancies with favorable safety profiles (40–42). However, research on the clinical application of ICIs in GC lags behind that of many other solid tumors. Nevertheless, the volume of literature in this field is steadily increasing, especially with regard to PD-1/PD-L1 inhibitors. Currently, several PD-1/PD-L1 inhibitors have been approved by the FDA for GC treatment, including Pembrolizumab, Atezolizumab, and Nivolumab, providing new therapeutic options for patients and laying the foundation for future research and applications in immunotherapy.

### 3.2 Application of PD-1/PD-L1 inhibitors in gastric cancer

PD-1 is a 55 kD transmembrane protein expressed on the surface of activated T cells, B cells, macrophages, dendritic cells (DCs), monocytes, and natural killer cells. Furthermore, PD-1 expression is significantly elevated on tumor-specific T cells and was identified in 1992 as a protein involved in the regulation of programmed T cell death (43). PD-L1, the ligand for PD-1, is widely expressed on the surface of activated T cells, B cells, macrophages, and DCs, and is also expressed in various tumor cells, including gastric cancer (44). When PD-1 on the surface of activated T cells binds to PD-L1 expressed on tumor cells, it inhibits T cell activation, proliferation, survival, secretion of T cell-associated cytokines, and cytotoxic activity, leading to impaired host anti-tumor immune responses and enabling tumor immune escape (45). Therefore, the theoretical basis for ICIs targeting PD-1/PD-L1 signaling pathway is to use PD-1 antibodies to bind PD-1 on activated T cells or anti-PD-L1 antibodies to bind PD-L1 on tumor cells, disrupting the interaction between PD-1 and PD-L1. This alleviates T cell immune tolerance and restores host anti-tumor immunity, enabling CD8+ effector T cells to regain the ability to kill tumor cells (46).

In the field of GC treatment, the clinical potential of PD-1/PD-L1 inhibitors is becoming increasingly prominent particularly PD-1 inhibitors such as pembrolizumab. In the 2016 KEYNOTE-012 trial, pembrolizumab demonstrated its ability to activate T cell responses in patients with advanced GC, with 8 out of 39 evaluable patients (22%) achieving partial response (47). Subsequently, in the KEYNOTE-059 trial, pembrolizumab monotherapy showed favorable antitumor activity and safety in patients with advanced GC or gastroesophageal junction (GEJ) cancer. These findings led the FDA to approve pembrolizumab in September 2017 as a third-line treatment for patients with locally advanced or metastatic GC or GEJ cancer patients with a combined positive score (CPS) ≥1. Furthermore, patients with PD-L1-positive tumors had higher objective response rates (ORR) and longer response durations compared to those with PD-L1-negative tumors when treated with pembrolizumab monotherapy (48). In addition, studies have reported that PD-L1 CPS can serve as an indirect predictor of OS in advanced GC patients receiving ICIs therapy. Both the KEYNOTE-059 and KEYNOTE-061 trials demonstrated that patients with PD-L1 CPS scores ≥1 or ≥10 were more likely to benefit from pembrolizumab treatment (49).

Another PD-1 inhibitor, nivolumab, has also demonstrated significant efficacy in GC patients. The CheckMate-649 trial, the largest global phase III clinical trial for advanced gastric cancer, evaluated the efficacy of nivolumab in combination with chemotherapy regimens—either oxaliplatin plus capecitabine (XELOX) or leucovorin, fluorouracil, and oxaliplatin (FOLFOX). Compared with chemotherapy alone, the combination therapy significantly prolonged median OS (13.8 months vs. 11.6 months), reducing the risk of death by 20%. The benefit was particularly pronounced in patients with PD-L1 CPS ≥5 (50). Subgroup analysis in Chinese patients showed results consistent with the global population, demonstrating that the combination therapy significantly improved OS in the PD-L1 CPS ≥5 population. Based on these findings, the FDA approved nivolumab plus chemotherapy in April 2021 for the first-line treatment of advanced or metastatic GC and GEJ cancer regardless of PD-L1 expression status. In 2022, the Chinese Society of Clinical Oncology (CSCO) included for the first time in its GC guidelines the recommendation that nivolumab combined with XELOX or FOLFOX be used as a first-line treatment for patients with HER2-negative advanced GC or GEJ cancer and a PD-L1 CPS ≥5. Therefore, PD-L1 expression in tumors has been widely adopted as a predictive biomarker to guide immunotherapy with PD-1/PD-L1 antibodies in advanced gastric cancer. Clinical guidelines have also raised the standards for the sensitivity and specificity of PD-L1 immunohistochemical testing (51). Accurate PD-L1 staining is essential for optimizing therapeutic decision and provides valuable guidance for clinical practice (**Table 2**).

**Table 2:** Application of PD-1/PD-L1 inhibitors in gastric cancer.

Study	Clinical effect	Conclusion
KEYNOTE-012	In patients with advanced GC, pembrolizumab activated T-cell responses, achieving an ORR of 22% (8/39) (47).	Provided initial proof of antitumor activity for pembrolizumab in advanced GC.
KEYNOTE-059	Pembrolizumab monotherapy showed favorable activity and safety in advanced GC/GEJ cancer (48).	Directly led to FDA approval (Sep 2017): as a third-line treatment for advanced GC/GEJ cancer with PD-L1 CPS ≥1.
KEYNOTE-059 & 061	PD-L1-positive (CPS ≥1/≥10) patients had significantly higher ORR and longer response duration compared to negative patients (48, 49).	Established PD-L1 CPS as a biomarker for predicting efficacy of pembrolizumab and patient overall survival (OS).
CheckMate-649	Nivolumab + Chemo (XELOX/FOLFOX) vs. Chemo alone: Significantly prolonged median OS (13.8 vs. 11.6 months), reducing death risk by 20% (50). Benefit was most pronounced in patients with PD-L1 CPS ≥5 (50).	1. FDA approval (Apr 2021): This combination for first-line treatment of advanced GC/GEJ cancer (regardless of PD-L1 status). 2. 2022 CSCO Guideline recommendation: as a first-line treatment for HER2-negative, PD-L1 CPS ≥5 patients.
Summary	PD-L1 expression level is clearly associated with the efficacy of PD-1/PD-L1 inhibitors.	1. PD-L1 is now a key biomarker guiding immunotherapy in advanced GC. 2. Clinical testing standards are raised; accurate PD-L1 testing is crucial for treatment decisions.

However, despite the promising clinical potential of immune checkpoint inhibitors, less than 40% of cancer patients are eligible for PD-L1/PD-1 antibody immunotherapy. Among treated patients, fewer than 20% achieve clinical survival benefits. Therefore, relying solely on PD-L1 expression is not the optimal biomarker for assessing the effectiveness of PD-L1/PD-1 blocking treatments in cancer patients (52, 53). There is still a need for more reliable biomarkers to predict and evaluate the efficacy and prognosis of PD-1/PD-L1 antibody immunotherapy, providing a scientific basis for clinicians to select appropriate treatments for various cancer patients. Existing studies suggest that H. pylori may play an important role in modulating cancer immunotherapy, especially PD-1/PD-L1 inhibitor therapy (54). However, the specific effects of H. pylori on PD-1/PD-L1 inhibitors remain inconsistent across studies.

Therefore, in-depth research on the impact and mechanisms of H. pylori on PD-1/PD-L1 inhibitor efficacy is essential. This will help identify the patient populations most likely to benefit from this therapy, thereby maximizing survival benefits. In the context of precision medicine, exploring Hp as a predictive biomarker will be a key direction for advancing the broad application of PD-1/PD-L1 antibody immunotherapy.

## 4 The impact of H. pylori infection on the efficacy of immunotherapy in gastric cancer and related mechanisms

### 4.1 The impact of H. pylori infection on the efficacy of immunotherapy in gastric cancer

Current studies suggest that H. pylori may play a significant role in modulating tumor immunotherapy, particularly ICIs therapy. However, the impact of H. pylori infection on the efficacy of ICIs therapy in GC patients remains controversial, with considerable discrepancies among reported findings (**Table 3**).

**Table 3:** The impact of H. pylori infection on the efficacy of immunotherapy in gastric cancer.

Viewpoint	Clinical efficacy
Associated with poor prognosis	1.In advanced GC patients receiving immunotherapy, H. pylori-positive individuals had significantly shorter PFS/OS and a lower response rate to anti-PD-1 therapy (55, 56). 2.H. pylori infection was an independent risk factor for shortened PFS (56).
Potentially beneficial effects	1.Large trials indicate that Asian patients (with higher H. pylori prevalence) gain more benefit from immunotherapy than Western patients (57). 2.A meta-analysis links H. pylori infection to higher tumor PD-L1 expression (58). 3.H. pylori-positive patients show longer PFS/OS and richer infiltration of active CD8+ T cells in the TME (59).

#### 4.1.1 Evidence for association with poor prognosis

Several studies have indicated that H. pylori infection may be associated with worse outcomes following ICI therapy. In metastatic GC patients receiving ICIs, H. pylori infection was associated with significantly shorter progression-free survival (PFS) and OS (55). Another analysis of advanced GC patients revealed a lower response rate to anti-PD-1 therapy in the H. pylori-positive group. Both PFS and OS were longer in the H. pylori-negative patients compared to those who were positive. Multivariate analysis further identified that H. pylori infection was an independent risk factor for shortened PFS, suggesting a potential negative association between H. pylori infection and immunotherapy outcomes in advanced GC patients (56).

#### 4.1.2 Evidence for potential beneficial effects

In contrast, other studies have suggested that H. pylori infection may enhance ICI efficacy. Basic research has shown that *in vitro* experiments using human gastric cancer cells demonstrated that H. pylori DNA vaccines can induce a shift from Th1 to Th2 responses, activate CD3+ T cells, and suppress tumor cell growth. Simulating the immune status of H. pylori-positive gastric cancer patients further revealed that abundant CD3+ T cells could inhibit the growth of gastric cancer xenografts *in vivo* (60). According to results from the randomized KEYNOTE-062 trial, the large international CheckMate-649 trial, and the randomized ATTRACTION-4 trial, Asian patients derived greater clinical benefit from anti-PD-1/PD-L1 therapies compared to patients in North America and Europe. Given the significantly higher prevalence of H. pylori infection in Asian GC patients than in Northern European and North American populations, it has been hypothesized that H. pylori infection may be a novel biological factor contributing to this regional difference in immunotherapy efficacy, potentially predicting better survival benefit from immunotherapy, particularly in Asian patients (57).

A meta-analysis including 1870 patients found that H. pylori infection was associated with tumor PD-L1 expression, suggesting it may serve as a potential predictive biomarker for favorable response to immunotherapy (58). Another study observed that H. pylori-positive patients had longer immune-related PFS and immune-related OS compared to H. pylori-negative patients. Mechanistically, the TME of H. pylori-positive patients exhibited higher PD-L1 expression levels and richer infiltration of non-exhausted CD8+ T cells, implying that H. pylori infection may favorably shape a “hot” TME conducive to GC immunotherapy (59).

In summary, current evidence regarding the influence of H. pylori infection on the efficacy of ICIs in GC is conflicting. To elucidate the relationship and underlying mechanisms, large-scale, well-designed prospective clinical studies are urgently needed. In-depth exploration of how H. pylori modulates immunotherapy responses and its mechanisms is crucial for optimizing GC immunotherapy strategies.

### 4.2 The mechanisms of H. pylori on gastric cancer immunotherapy

#### 4.2.1 H. pylori modulates PD-L1 expression levels to influence immunotherapy efficacy

Relevant studies have shown that elevated PD-L1 expression on tumor cells can impair the body’s anti-tumor immune response, inhibit the cytotoxic activity of T cells, and promote tumor progression. Research indicates that H. pylori and disease progression correlate positively with PD-L1 expression (61). Furthermore, it has been reported that PD-L1 expression is higher in H. pylori-positive GC patients compared to H. pylori-negative patients (62). Additionally, H. pylori infection can upregulate PD-L1 expression in the gastric mucosal cells of GC patients, thereby affecting the efficacy of immunotherapy (31).

Further mechanistic exploration shows that H. pylori can upregulate PD-L1 expression induced by its virulence factors. Holokai et al. demonstrated that H. pylori-induced PD-L1 expression in gastric epithelium during infection is mediated by the Sonic hedgehog (Shh) signaling pathway. H. pylori CagA protein can activate the Shh pathway via early activation of relevant proteins in parietal cells, resulting in increased PD-L1 expression (63). Moreover, H. pylori CagA regulates PD-L1 expression through the CagA-p53-miR34a-PD-L1 signaling axis, which suppresses CD8+ T cell proliferation and cytokine secretion, thus modulating immunotherapy (64). T4SS components of H. pylori activate the p38 MAPK pathway and upregulate PD-L1 expression, thereby inhibiting T cell proliferation and inducing the differentiation of regulatory T (Treg) cells from naive T cells, leading to immune escape (65). The urease B subunit of H. pylori induces high PD-L1 expression in bone marrow-derived macrophages (BMDMs) through myosin heavy chain 9 (Myh9) or mTORC1 signaling, significantly inhibiting CD8+ T cell proliferation and activation (66). Additionally, studies have observed the potential impact of lipopolysaccharide (LPS) on PD-L1 expression in GC cells. LPS stimulation significantly increased PD-L1 expression in GC cells. Furthermore, nuclear factor-κB (NF-κB) activation was involved in PD-L1 expression in GC cells exposed to LPS stimulation through p65 binding to the PD-L1 promoter. H. pylori LPS activates NF-κB, which promotes PD-L1 expression in GC cells (67). Small extracellular vesicles (sEVs) derived from H. pylori-positive GC cells promote lymphangiogenesis and lymphatic remodeling by transferring overexpressed miR-1246 from sEVs to lymphatic endothelial cells, resulting in lymphocyte proliferation and stabilizing PD-L1 expression (68). Arnold et al. reported that in H. pylori-infected murine gastric tissue, recruited gastrointestinal eosinophils were activated by direct bacterial contact. This led to an IFN-γ-dependent upregulation of PD-L1, thereby suppressing Th1/Th17 responses and T cell proliferation via the PD-1/PD-L1 axis to facilitate immune escape (69).

In conclusion, H. pylori may affect PD-L1 expression through multiple pathways, including Shh, CagA-p53-miR34a-PD-L1, p38 MAPK, Myh9/mTORC1, NF-κB, sEVs, and IFN-γ, thereby influencing the efficacy of immunotherapy.

#### 4.2.2 H. pylori modulates immunotherapy efficacy by influencing the tumor microenvironment

The TME is a complex composite made up of tumor cells and an evolving stroma, which includes surrounding non-cancerous cells (such as fibroblasts, epithelial cells, immune cells, and blood cells) as well as extracellular components (such as cytokines, growth factors, hormones, and extracellular matrix) (70, 71). The TME plays a critical role in tumor initiation, progression, and metastasis, and it significantly influences treatment response and clinical outcomes (72). The high heterogeneity of GC may be one of the key factors contributing to the poor efficacy of immunotherapy, highlighting the necessity of in-depth exploration of the various cellular and molecular mechanisms within the tumor immune microenvironment.

TLS is an ectopic lymphoid aggregates formed in non-lymphoid tissues. It enhances antigen presentation through B cell clonal expansion, drives cytotoxic immune responses, and promotes humoral immunity. In the TME, the TLS plays a crucial role in the complex interplay between immune and tumor cells by recruiting and activating naive T and B cells through chemokine signals (73). Recent studies indicate that the presence of TLS is associated with improved prognosis in various gastrointestinal cancers. Specifically in gastric cancer, the TLS and B cell infiltration are strongly correlated with favorable patient responses to anti-PD-1 therapy (74–77). Consequently, the presence of TLS in the TME often predicts longer patient survival and enhanced responsiveness to ICIs. Emerging evidence suggests that the gut microbiota can promote TLS formation. For instance, the colonization of H. pylori within tumors can induce the development of TLS, thereby triggering an anti-tumor immune response (78). Concurrently, H. pylori infection helps shape an immune cell-rich, “hot” TME, which is significantly associated with TLS (59, 79). Therefore, H. pylori can modulate therapeutic responses to ICIs by promoting the formation of TLS and shaping the “hot” TME, which demonstrates its significant potential in tumor immune regulation.

H. pylori and its virulence factors influence the composition and function of the TME, such as tumor-associated macrophages (TAMs), bone marrow mesenchymal stem cells (BM-MSCs), cancer-associated fibroblasts (CAFs), and myeloid-derived suppressor cells (MDSCs), shaping a microenvironment favorable for its own survival and colonization. This modulation plays a pivotal role in GC development, progression, and immune response (16). Given the central role of the TME in GC progression and immune regulation, TME immunotherapy strategies have become a major focus of recent anti-cancer clinical research, showing significant clinical application prospects and development potential.

H. pylori and TAMs are closely related to changes in immune responses, which are emerging as key players in the TME. TAMs play an important role in host resistance to bacterial infections and in regulating the body’s immune response (80). H. pylori can modulate TAM differentiation, disrupt the M1/M2 balance, and favor the M2 phenotype to evade immune surveillance (81). H. pylori infection can regulate macrophage function by modulating specific microRNAs, such as upregulating the expression levels of let-7i-5p, miR-146b-5p, and miR-185-5p (82, 83).

Multipotent mesenchymal stem cells (MSCs) can self-renew and differentiate into various cell types, playing a key role in tissue healing, regeneration, and immune regulation (84). BM-MSCs may be associated with gastric tumorigenesis and immune suppression. Upon sensing signals indicating gastric mucosal damage, BM-MSCs migrate from the bone marrow to the stomach via peripheral circulation. BM-MSCs repair the damaged mucosa through paracrine mechanisms and directed differentiation (85, 86). Chronic inflammation induced by H. pylori infection recruits bone marrow-derived MSCs to the stomach, where they undergo malignant transformation due to clonal dominance. BM-MSCs can also reduce the proportion of IFN-γ-producing T cells, inhibit the proliferation of CD4+ and CD8+ T cells, leading to local and systemic immune suppression and H. pylori-induced GC (87).

CAFs are activated myofibroblasts commonly found in solid tumors and constitute a major component of the tumor stroma, which plays significant roles in the TME. CAFs can create a niche for cancer cells and promote cancer progression by stimulating tumor cell proliferation, migration, invasion, and angiogenesis (88, 89). The various pro-inflammatory and tumor-associated factors secreted by CAFs may induce chronic inflammation and significantly modulate tumor immunity, thereby facilitating immune escape (90). H. pylori infection can induce the differentiation of MSCs into CAFs and upregulate the expression of fibroblast markers, fibroblast activation protein (FAP), CAF activation markers, and invasive markers (91). FAP-positive CAFs enhance the survival, proliferation, and migration of GC cell lines and significantly inhibit T cell function and proliferation (92). Critically, H. pylori-induced CAFs, particularly FAP-positive CAFs, are key contributors to the reduced efficacy of ICIs. Therefore, H. pylori infection-driven activation and expansion of CAFs shape a highly immunosuppressive TME that excludes T cells. This severely limits the ability of ICIs to reactivate anti-tumor immune responses and represent a major mechanism of immunotherapy resistance.

MDSCs are key immune-suppressive cell populations in the TME, mediating potent immune suppression through various mechanisms. The high abundance of MDSCs in H. pylori infected patients is associated with advanced GC and poor prognosis (93, 94). H. pylori-induced MDSC infiltration is a key driver of gastric immune suppression, immune dysfunction, and gastric tumorigenesis. Importantly, this MDSC-mediated profound immune-suppressive state significantly reduces the efficacy of chemotherapy and immunotherapy (95). H. pylori can induce bone marrow cells to differentiate into MDSCs with specific phenotypes, such as SLFN4+ MDSCs, which have stronger immune suppressive abilities and can effectively inhibit T cell function, leading to immune dysregulation and promoting tumor progression (96). Therefore, H. pylori-driven expansion and activation of MDSCs can construct a microenvironment that deeply suppresses T cell function, promotes immune suppression networks, and enhances adaptive immune resistance. This is one of the key mechanisms underlying the low response rates or resistance to ICIs in GC patients.

Therefore, in the TME of GC, promoting the formation of TLS or inhibiting the immune suppressive pathways mediated by TAMs, MSCs, CAFs and MDSCs is expected to become a potential strategy for improving the efficacy of immunotherapy for GC.

#### 4.2.3 H. pylori modulates gut microbiota to influence immunotherapy

In the past few decades, various microbiomes have been shown to participate in the development and progression of different cancers, which have significant clinical implications for tumor diagnosis and treatment. Microbiome transplantation, immunotherapy, probiotics, microbe-targeted tumor therapy, postbiotic therapy, and antibiotic therapy have made significant progress, providing an important theoretical foundation for microbiome-targeted cancer treatment (97). Among these, the gut microbiota plays a key role in modulating cancer immunotherapy, particularly the efficacy of ICIs (54). The gut microbiota is essential for both innate and adaptive immunity in the host. Its balance helps establish the immune system and enhances the efficacy of ICIs. Conversely, its disruption impairs immune function, leading to low immune responses and reduced efficacy of ICIs. Sivan et al. found that differences in gut microbiota environments in C57BL/6 mice could result in significant differences in melanoma immunotherapy. Bifidobacterium was found to strongly induce tumor-specific T cell immunity, suggesting that bifidobacteria could act as an immune enhancer for anti-PD-L1 antibody therapy (98). Routy et al. demonstrated the impact of the gut microbiota on immunotherapy in a group of patients with NSCLC, renal cell carcinoma, or urothelial carcinoma. Patients treated with antibiotics had significantly lower PFS and OS, indicating that gut microbiota dysbiosis could reduce the efficacy of immunotherapy (99–101).

Studies have shown that H. pylori infection can alter the composition of the gut microbiota in both human and animal models, thereby influencing the efficacy of immunotherapy (102, 103). Increasing the proportion of probiotics can activate cell-mediated immune responses by inducing Batf3-lineage DCs and Th1 cells, while reducing the proportion of certain bacteria can decrease FoxP3+ Treg cell induction, thereby enhancing anti-tumor effects of ICIs (101). The gut microbiota may break immune tolerance and reactivate the host immune response, thus enhancing the efficacy of PD-1/PD-L1 inhibitor therapy. Recently, many clinical trials on fecal or probiotic transplantation combined with PD-1/PD-L1 inhibitors have been conducted, showing encouraging results and holding potential to become a major trend in future PD-1/PD-L1 inhibitor therapy (104). A study found that the traditional Chinese medicine formula Weitiao No. 3 mixture might enhance the efficacy of GC immunotherapy by increasing the abundance of Bifidobacterium and Faecalibacterium in the gut microbiota, raising levels of isobutyrate and isovalerate, and thereby modifying the gut microbiota (105). However, it remains unclear whether H. pylori infection can affect the proportion of probiotics within the gut microbiota. More evidence is needed to clarify and confirm this issue.

The gut microbiota and its metabolites not only regulate the function of inflammatory and immunosuppressive cells but also influence the formation of TLS (106). Classic microbial metabolites, such as short-chain fatty acids (SCFAs), bile acids (BAs), and tryptophan (Trp) (107–110), can enter systemic circulation and act on the TME. Among these, SCFAs can enhance T cell activation and promote M1 macrophage polarization, thereby improving the efficacy of PD-1/PD-L1 blockade therapy (111). In gastric cancer, the microbial-derived butyric acid can further suppress the expression of immune suppressive factors like PD-L1 and IL-10 in tumor-associated macrophages (112). Besides metabolites, the gut microbiota itself can also translocate to tumor tissues through the circulatory system and participate in the construction of TLS (113). It is worth noting that the colonization of H. pylori has been shown to induce TLS formation within the gastric cancer microenvironment, thereby enhancing the response to ICIs (79). In summary, the gut microbiota and its metabolites regulate the local immune microenvironment through multiple pathways, promote TLS formation, and ultimately influence the efficacy of immunotherapy.

Therefore, when exploring the correlation between H. pylori and ICI efficacy, it is important to also consider the role of the gut microbiota. In the future, the regulation of the gut microbiota holds great promise for the development of GC immunotherapy. On one hand, in-depth studies on the relationship between the gut microbiota and GC immunotherapy may reveal new therapeutic targets and mechanisms, thereby enhancing treatment efficacy. On the other hand, modulating the gut microbiota and its metabolites could enable personalized optimization of immunotherapy regimens for GC patients, enhancing treatment specificity and safety.

In summary, H. pylori infection may impact immunotherapy efficacy and anti-tumor responses through multiple regulatory pathways. H. pylori infection modulates PD-L1 expression in host cells through multiple pathways, including Shh, CagA-p53-miR34a-PD-L1, p38 MAPK, Myh9/mTORC1, NF-κB, sEVs, and IFN-γ, thereby influencing immune responses. Moreover, H. pylori and its virulence factors affect the composition of the TME and promote the formation of TLS to regulate GC development, progression, and immune responses. Additionally, H. pylori infection can alter the gut microbiota, further modulating immune responses. Therefore, H. pylori infection status should be considered when applying ICIs to optimize immunotherapy outcomes (**Table 4**).

**Table 4:** The mechanisms of H. pylori on gastric cancer immunotherapy.

Mechanism	Roles of H. pylori	Result
PD-L1 Expression	CagA: Upregulates PD-L1 through the Shh pathway and the CagA-p53-miR34a-PD-L1 axis (63, 64). T4SS: Activates the p38 MAPK pathway to upregulate PD-L1 (65). Urease B: Induces high expression of PD-L1 in macrophages through the Myh9/mTORC1 pathway (66). LPS: Promotes PD-L1 expression by activating NF-κB (67). sEVs: sEVs transfer miR-1246, stabilizing PD-L1 expression (68). Eosinophils: Upregulates PD-L1 through the IFN-γ pathway (69).	Upregulates PD-L1 through multiple pathways, the function and proliferation of T cells are inhibited, thereby affecting the efficacy of immunotherapy.
TME	1. Promotes TLS formation, helping create the “hot” TME (59, 78, 79). 2. Modulates immunosuppressive cells: TAMs: Shifts balance toward M2 phenotype, regulates miRNAs (81–83). BM-MSCs: Recruited to the stomach to inhibit T cell function, resulting in immunosuppression (85–87). CAFs: Induces differentiation (especially FAP^+^CAFs), which inhibit T cells and promote tumor progression (91, 92). MDSCs: Induces the differentiation (such as SLFN4^+^ MDSCs), and inhibit the function of T cells (93–96).	Dual modulation: TLS induction may improve ICI response; conversely, activation of TAMs, CAFs, and MDSCs creates a highly immunosuppressive TME that excludes T cells and drives ICI resistance.
Gut Microbiota	Affects the proportion of probiotics and thereby influencing immunotherapy (98). The microbiota and its metabolites can regulate the function of immune cells and promote the formation of TLS, thereby influencing immunotherapy (106–112).	The gut microbiota, its metabolites, and the proportion of produced probiotics can all affect immunotherapy. Regulating the gut microbiota is a potential strategy to enhance the GC immunotherapy.

## 5 Summary and prospects

This review summarizes the impact of H. pylori infection on the development, progression, and immunotherapy of GC. Immunotherapy, particularly ICIs, has shown great potential in treating various types of tumors. However, ICIs primarily target specific ICs such as PD-1/PD-L1, and a considerable proportion of GC patients show limited response or develop resistance to these therapies. Among the various influencing factors, the expression level of PD-1/PD-L1 is one of the key determinants of ICI efficacy in GC. Notably, H. pylori infection introduces both new opportunities and challenges in this field. Substantial clinical evidence suggests a correlation between H. pylori infection and the efficacy of immunotherapy in cancers such as colorectal cancer, NSCLC, and melanoma. However, the precise relationship between H. pylori infection and ICIs therapy efficacy in GC patients remains unclear. Furthermore, H. pylori infection is a recognized cause of several diseases, with its association being most closely linked to gastric MALT lymphoma.

H. pylori infection may influence the human immune system and the antitumor response to immunotherapy, though the exact mechanisms remain unclear and warrant further investigation. Current evidence suggests that H. pylori can regulate PD-L1 expression in host cells through multiple pathways, including Shh, CagA-p53-miR34a-PD-L1, p38 MAPK, Myh9/mTORC1, NF-κB, sEVs, and IFN-γ, thereby influencing immune responses. In addition, H. pylori can influence the immune response through promoting the formation of TLS or affecting the composition and function of the TME through the modulation of TAMs, MSCs, CAFs and MDSCs. Moreover, H. pylori infection can lead to alterations in the gut microbiota and its metabolites, which further modulate host immune responses.

In summary, the specific role and clinical value of H. pylori in GC immunotherapy remain under investigation. Further basic and clinical studies are needed to elucidate its mechanisms and assess its therapeutic potential. Currently, therapeutic strategies targeting H. pylori-associated immune pathways are still limited, and more research is required to evaluate the predictive value of H. pylori infection status in determining responses to immunotherapy in GC patients. As this research deepens, we anticipate providing more effective immunotherapy strategies for GC patients, tailoring optimal treatment regimens based on individual characteristics to improve patient prognosis and quality of life.
